# The effect of low-intensity exercise on emotional and cognitive engagement in the classroom

**DOI:** 10.1038/s41539-022-00125-y

**Published:** 2022-05-26

**Authors:** Ayame Tamura, Kou Murayama, Ryo Ishii, Michiko Sakaki, Ayumi Tanaka

**Affiliations:** 1 University of Human Environments, 6-2 Motojuku-cho Sanbonmatsu, Okazaki-shi, Aichi, 444-3505 Japan; 2 University of Tübingen, Europastraße 6, 72072 Tübingen, Germany; 3 University of Reading, Whiteknights, PO Box 217, Reading, RG6 6AH United Kingdom; 4 Nara University of Education, Takabatake-cho, Nara-shi, Nara, 630-8528 Japan

**Keywords:** Human behaviour, Education

## Abstract

This study examined whether engaging in physical exercise during a university class would have beneficial effect on students’ learning motivation. One hundred and forty-nine participants took part in a psychology class over nine weeks (one lesson per week); for each lesson, participants engaged in a three-minute physical activity (low-intensity aerobic exercise) or control activity (watching a video), about 20 min after the lesson started. Participants reported higher vigour and lower fatigue during the class when they exercised than when they engaged in control activities. These findings suggest the utility of incorporating a short exercise activity in university settings to enhance students’ classroom motivation.

## Introduction

A number of previous studies have found that physical activities have beneficial effects not only on physical health^1,2^ but also on mental health^3–5^. However, less is known about their beneficial effect in educational settings. Previous studies have examined the effects of exercise on academic performance, but their results have been inconclusive^6,7^. For example, in reviewing 50 studies on the relationship between school-based physical activity and academic performance, Rasberry et al. concluded there was no consistent link between physical activity and academic performance^8^. The results from recent meta-analyses also reported mixed findings; some showed small but overall positive effects^9–11^, while others indicated that the overall effect was non-existent and that the data were inconsistent^12–14^. Thus, there is no consensus on whether exercise truly has a significant impact on academic outcomes.

To gain a better understanding of these inconsistent findings, it is important to examine the potential mechanisms underlying the effects of exercise on academic performance^15^. This study aimed to examine the effect of exercise on a key predictor of academic achievement—motivation^16^. There has been little research that systematically examines the influence of exercise on motivation in educational settings. In this study, we focused on emotional and cognitive engagement^17^ as indices of classroom motivation and tested the hypothesis that a physical exercise intervention during a university lecture would positively influence emotional and cognitive engagement.

Several studies have shown that exercise improves mental health and lowers stress, anxiety, and depression^3–5^. Previous studies have demonstrated that exercise affects more general affective states, i.e., mood; for instance, research examining mood changes after physical activities reported decreased negative mood, tension, and fatigue, whereas mood and vigour were improved^18,19^. Such beneficial effects are often explained by physiological changes caused by exercise. Specifically, previous studies have repeatedly demonstrated that engaging in physical exercise triggers changes in neurotransmitter levels, especially monoamines^5^, which in turn helps decrease negative moods and increase positive moods^20,21^. For example, in studies on rodents, physical activity modulates levels of norepinephrine in the locus coeruleus, amygdala, and prefrontal cortex^22,23^, and leads to activity in the dorsal raphe nucleus serotonin neuron^24^. Similar findings have been also observed in humans. For instance, it is well documented that plasma norepinephrine concentrations increase after exercise in humans^25,26^. In addition, in a randomised controlled study on aerobic exercise^27^, individuals who completed a 7-week exercise intervention showed lower levels of depression and anxiety, as well as a larger change in serum serotonin levels, compared with those who completed a control-stretching intervention. Building on these past findings, we expected students’ general mood (i.e., vigour, fatigue and depression) as well as their subjective feeling of interest, a key emotional aspect in academic engagement^28,29^, to improve by incorporating exercise during class.

Increased norepinephrine levels due to exercise may also result in stronger physiological arousal and better cognitive functioning^6,11,30^. Norepinephrine is primarily sourced from the locus coeruleus in the brain and has been implicated in modulating alertness and arousal^31^. The locus coeruleus also projects throughout the brain^30,32^. Therefore, when the locus coeruleus is activated due to exercise, it will result in widespread norepinephrine release including in the prefrontal cortex, which in turn enables individuals to better maintain attention to task-relevant stimuli while prevent attention to distractors when norepinephrine levels are high^32,33^. In line with this idea, there is a large body of literature that suggests exercise affects cognitive functions, such as attention^30,34^. Such beneficial effects of exercise on cognitive functioning may be observed even in educational settings. However, despite the obvious importance of attention in academic learning^17,35,36^, few studies have examined the potential positive effects of physical exercise on learners’ attention in educational settings. Therefore, in the present study, we examined the effects of exercise on cognitive engagement.

In this study, cognitive engagement was assessed using two indices: Mind-wandering and sleepiness. Mind-wandering, also known as “task-unrelated thought”, is defined as a decoupling of attention from an external stimulus to internal thoughts^36^, and is considered one behavioural aspect that marks attention lapses^37^. Mind-wandering is relatively frequent in educational settings (e.g., during lectures^38^), and increased mind-wandering during lessons has been negatively associated with learning lecture materials^38^. Similarly, sleepiness during learning activities can be considered the consequence of attention lapses, and many university students experience excessive daytime sleepiness^39,40^. Previous studies showed that daytime sleepiness was associated with poor academic performance^39^ and motivation^40^. In light of previous studies showing that physical activity improves cognitive or attentional processing in controlled lab experimental studies^41–43^, we expected these effects to also be observed in educational settings; we expected that exercise would decrease mind-wandering and sleepiness during class.

In the current study, we chose the type of physical activity deemed most relevant, practical, and useful in a classroom setting. Specifically, we chose a short duration (e.g., 3 min) ‘slow aerobics’ programme developed by experts^44^ as the target exercise activity in our intervention. Slow aerobics was originally developed as a ten-minute programme combining upper limb, lower limb and trunk movements with a low-intensity exercise that does not cause participants’ average heart rate to exceed 110 bpm; this is considered a low-intensity programme for young adults^44^. Given that the effects of exercise can be enhanced when accompanied by music^44^ or made into a group activity^45^, these elements are also included in the slow aerobics programme^44^. As exercise intensity is negatively associated with long-term adherence, relatively low-intensity exercise routines are easier to maintain^46^. In addition, low-intensity exercise routines can be easily practised even by people with relatively low physical strength^44^. Importantly, while many studies have reported the benefits of medium- and high-intensity exercise^20^, recent evidence shows that low-intensity exercise also enhances arousal^30^ and has beneficial effects on mood^47^ and cognitive function^30^. A shorter three-minute programme has also been developed to encourage reasonable and habitual exercise^44^, and this study adopted a three-minute version in consideration of its applicability in educational settings.

To examine whether low-intensity exercise during lessons can affect students’ motivation in an educational setting, nine lessons from a semester-long university class in psychology were randomly assigned to the exercise condition (four lessons) and control condition (five lessons). Twenty minutes after the class started, participants performed 3 min of slow aerobics in the exercise condition while watching a video displaying an underwater scene in the control condition. The class resumed immediately after this brief intervention period. To evaluate the potential benefit of exercise during class, we assessed participants’ self-reported motivation after each class (i.e., vigour, fatigue, depression, and interest as emotional engagement, as well as mind-wandering and sleep frequency as cognitive engagement).

## Results

### Descriptive statistics and correlation

**Fig. 1:**
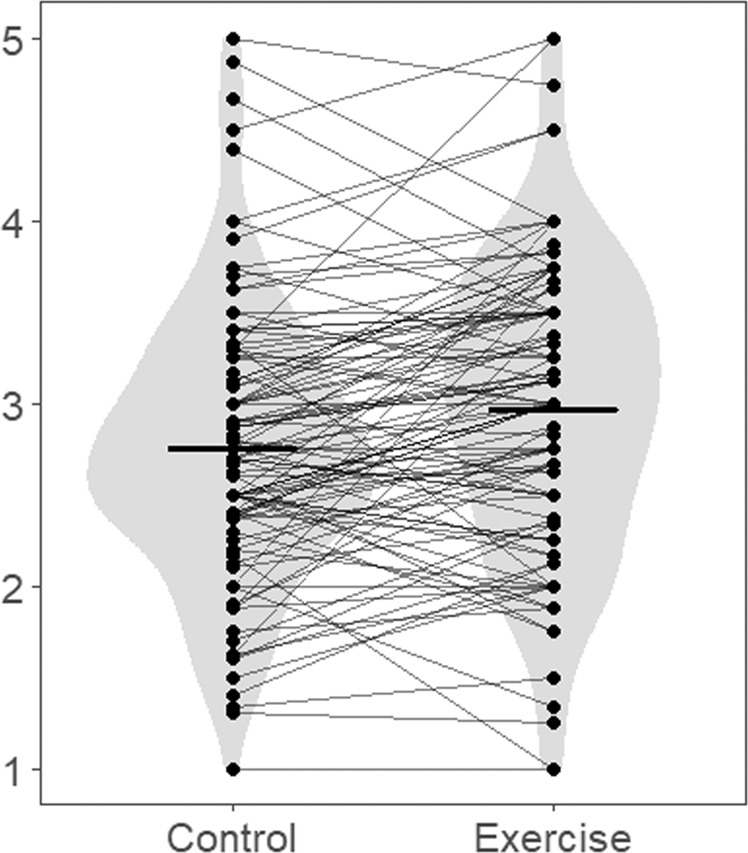
Violin plot for means within individuals and means between conditions of vigour in the exercise and control conditions. The dots represent the means within the individual, and the bars represent the means between the conditions.

**Fig. 2:**
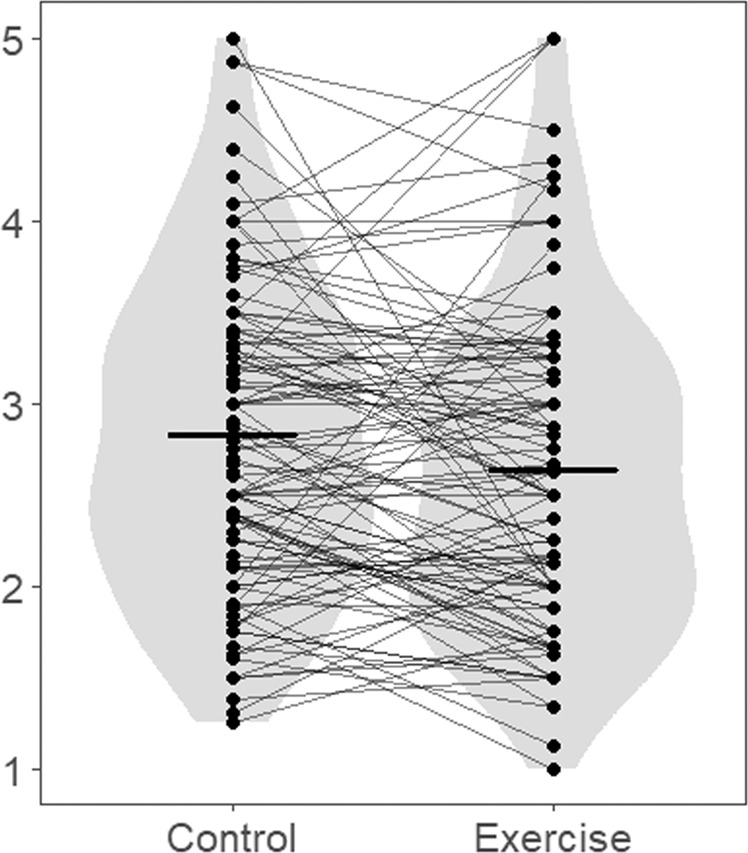
Violin plot for means within individuals and means between conditions of fatigue in the exercise and control conditions. The dots represent the means within the individual, and the bars represent the means between the conditions.

We calculated descriptive statistics for each lesson (Table 1). Figures 1 and 2 present violin plots for means within individuals and means between conditions of some variables (i.e., vigour and fatigue), where the effects of exercise were observed in the mixed-effects modelling described below. Within-person-level correlations and Cronbach’s alphas are reported in Table 2. To calculate correlations and Cronbach’s alphas between these within-person variables, we removed between-person variations according to the formula suggested by Kenny and La Voie^48^. As Table 2 shows the correlations between all variables, except the association between depression and sleep frequency, were low to moderate. Particularly, vigour had a significant positive correlation with interest and a significant negative correlation with fatigue, depression, sleep frequency and mind-wandering at the within-person level. This meant that as individuals felt more vigour, they also felt more interest and experienced less fatigue, depression, sleepiness, and mind-wandering. Similarly, fatigue had a significant positive correlation with sleep frequency and a significant negative correlation with interest at the within-person level. Depression had a significant positive within-person-correlation with mind-wandering and a significant negative within-person-correlation with interest. Interest had a significant negative correlation with sleep frequency and mind-wandering, and sleep frequency had a significant positive correlation with mind-wandering at a within-person level.

**Table 1:** Descriptive statistics of variables.

Variable	Week																	
	1		2		3		4		5		6		7		8		9	
	*M*	SD	*M*	SD	*M*	SD	*M*	SD	*M*	SD	*M*	SD	*M*	SD	*M*	SD	*M*	SD
Vigour	2.82	0.94	3.04	1.02	2.79	1.04	3.01	0.90	2.69	0.93	2.76	0.97	2.86	1.04	2.97	1.00	2.56	0.99
Fatigue	2.90	1.13	2.60	1.02	2.73	1.19	2.65	1.15	2.72	1.07	2.35	1.06	2.61	1.02	2.66	1.15	2.94	1.23
Depression	1.69	1.00	1.39	0.56	1.49	0.79	1.48	0.83	1.58	0.85	1.40	0.74	1.31	0.49	1.45	0.71	1.56	0.86
Interest	5.33	0.93	5.44	0.96	5.17	1.22	5.24	1.03	5.05	1.10	5.12	0.99	5.17	0.98	5.08	1.09	4.79	1.10
Sleep frequency	0.18	0.53	0.22	0.55	0.54	0.78	0.22	0.60	0.26	0.69	0.35	0.75	0.41	0.82	0.19	0.48	0.48	1.08
Mind-wandering	4.00	2.06	3.50	1.84	3.85	2.29	4.05	2.31	3.52	1.74	3.76	2.19	3.64	1.92	3.54	1.96	3.93	2.25
Condition	Control		Exercise		Control		Exercise		Control		Exercise		Control		Exercise		Control	
*n*	114		106		114		114		113		111		111		114		113	

*Note*. The range of scores was as follows: vigour, fatigue and depression were 1–5, interest was 1–7, sleep frequency was 0–6, and mind-wandering was 1–10.

**Table 2:** Within-person-level correlations.

	1	2	3	4	5	6	Alpha
1. Vigour	–	−0.39 (0.00)	−0.24 (0.00)	0.49 (0.00)	−0.31 (0.00)	−0.30 (0.00)	0.87
2. Fatigue		–	0.24 (0.00)	−0.32 (0.00)	0.29 (0.00)	0.17 (0.00)	0.83
3. Depression			–	−0.17 (0.00)	0.06 (0.09)	0.08 (0.03)	0.78
4. Interest				–	−0.28 (0.00)	−0.31 (0.00)	0.80
5. Sleep frequency					–	0.08 (0.03)	–
6. Mind-wandering						–	–

*Note*. The two-tailed *P* value is shown in parentheses.

### Overview and preliminary analysis

This study employed a within-subjects design with repeated assessments over nine lessons: four lessons were randomly assigned to the exercise condition and five were assigned to the control condition. We chose to conduct linear mixed-effects modelling that effectively compared the conditions by taking into account the missing data (i.e., the participants who only partially participated in the sessions), time/lesson effects and the difference in the number of lessons assigned to the exercise condition (four lessons) and control condition (five lessons). Note that some of the variables were not normally distributed (e.g., depression, interest, sleep frequency, and mind-wandering), but previous research has suggested that mixed-effects modelling is robust for the violation of distributional assumptions^49^. Each model predicted the dependent variable (e.g., sleep frequency) from the exercise condition (exercise = 1, control = −1) as a fixed effect. We also included the linear time effect (coded from 0 to 8, representing the assessment week) as well as its random slopes of participants to control for an overall time-dependent change^50^. Linear time trends were mean-centred variables.

Before the main analysis of linear mixed-effects models, we examined whether there were statistically significant random intercepts of lessons for each dependent variable by comparing models with and without the random intercepts. We included random intercepts of participants and lessons, as well as random participant slopes of the exercise condition. Random intercepts of the lessons were incorporated to account for a potential lesson-specific effect (e.g., one lesson happened to be more engaging than another lesson)^50^, representing the natural between-lesson variation of emotional and cognitive engagements. Random intercepts of the lessons served as a nuisance factor when statistically comparing the exercise and control conditions, and the inclusion of such a variance component (when it existed) ensured the generalisability of the findings beyond the lessons selected. In the models predicting sleep frequency, a significant difference was observed between those with and without the random intercept of lessons (*χ*^2^ = 6.80, df = 1, *P* < 0.01). There was no other model with significant random intercepts of lessons (vigour: *χ*^2^ = 1.36, df = 1, *P* = 0.24, fatigue: *χ*^2^ = 1.70, df = 1, *P* = 0.19, depression: *χ*^2^ = 2.45, df = 1, *P* = 0.12, interest: *χ*^2^ = 0.00, df = 1, *P* = 1.00, mind-wandering: *χ*^2^ = 1.04, df = 1, *P* = 0.30). As random intercepts of lessons often led to convergence errors, random intercepts of lessons were added only to the model predicting sleep frequency, not those predicting other dependent variables, as the variance estimates were not statistically significant. These analytic steps can effectively exclude the possibility that intervention effects simply reflect the ideocratic difference of class contents or learning materials^50^.

### Main analysis: examining the effects of exercise

In the main analysis of the linear mixed-effects models, we addressed the convergence issues as follows. If the model did not converge or if there was a singularity warning, we first removed the covariances among the random effects and then the random intercepts (because we were interested in the slope of the exercise condition) to aid model convergence or solve the singularity problem. According to Brauer and Curtin^51^, this fix could still provide reliable parameter estimates and standard errors for the exercise condition’s effect. If the model still did not converge or if a singularity warning appeared (e.g., the random effect was too small), then the random effects with corresponding variables were removed.

Supplementary Table 1 presents the original model (before addressing the convergence problem). We removed the covariance of the random effect for the time trends in the models that predicted fatigue, depression, and sleep frequency, where the covariance between the random effect for the exercise/control condition and random intercept still remained. In addition, we removed the random effect of the condition in the models that predicted vigour and interest because the singularity problem remained, even if the covariances of the random effect and random intercept were deleted. For the model that predicted mind-wandering, the random effects of both the condition and time trends were removed for the same reason as for vigour and interest. Only the random intercept remained in this model. Importantly, there were no differences in the significance of the effect of the condition on each dependent variable between the original and final models. Thus, we believed the final model could still provide reliable parameter estimates and standard errors for the exercise condition’s effect.

Table 3 shows the results obtained from the final model. The exercise intervention showed a significant positive effect on vigour, indicating participants felt more vigorous when they exercised during class than when they did a control activity (*B* = 0.10, *t* = 3.85, *P* < 0.001). Furthermore, the intervention also had a significant negative effect on fatigue (*B* = −0.09, *t* = −2.61, *P* < 0.05). The result showed that participants experienced less fatigue when they exercised during class, compared with the time they were engaged in control activities. The condition effects were not significant in the models predicting depression, interest, sleep frequency, and mind-wandering. The effect of the time trends was not significant except for the model that predicted interest (*B* = −0.06, *t* = −4.82, *P* < 0.001), which indicates that the participants experienced less interest over time (i.e., as the lessons progressed).

**Table 3:** Results of linear mixed-effects models with the final model addressing the convergence and singularity problems.

	Vigour			Fatigue			Depression			Interest			Sleep frequency			Mind-wandering		
	*B*	*t*	*P* value	*B*	*t*	*P* value	*B*	*t*	*P* value	*B*	*t*	*P* value	*B*	*t*	*P* value	*B*	*t*	*p* value
Intercept	2.85	43.38	0.00	2.72	37.87	0.00	1.54	29.41	0.00	5.16	69.84	0.00	0.30	5.62	0.00	3.77	28.34	0.00
Condition	0.10	3.85	0.00	−0.09	−2.61	0.01	−0.04	−1.77	0.08	0.04	1.61	0.11	−0.06	−1.40	0.20	−0.05	−0.79	0.43
Time	−0.02	−1.70	0.09	0.00	0.03	0.97	−0.01	−1.33	0.19	−0.06	−4.82	0.00	0.02	1.08	0.32	−0.01	−0.36	0.72

*Note*. The code of the condition was exercise condition = 1 and control condition = −1. *P* value = two-tailed *P* value.

## Discussion

This study aimed to examine the influence of low-intensity exercise on a variety of emotions (e.g., vigour, fatigue, depression, and interest) and cognitive engagements (e.g., sleep frequency and mind-wandering) in university classrooms. The results demonstrated that exercise had a positive effect on emotional engagement variables, specifically moods, such as increased vigour and decreased fatigue, suggesting the beneficial effect of exercise on the emotional aspects of academic motivation. It is worth noting that the current study used a short (only 3 min) low-intensity aerobic exercise, which is easy to conduct in a wide range of contexts. The exercise programme we adopted had a lower intensity and shorter duration than other successful exercise programmes in educational settings^15,52^.

The results of our study showed that the laboratory findings observed in previous studies can apply to a real classroom context. The participants in the present study were in positive moods during the lessons in the exercise condition. The next logical step is to extend the study to examine the potential beneficial effects of physical exercise on academic achievement mediated by emotional engagement (i.e., positive mood). Importantly, the current findings may provide a clue of the types of achievements that physical exercise could facilitate. For example, a previous study reported that inducing a positive mood enhances the performance of a category learning task associated with cognitive flexibility^53^. Therefore, exercise may have positive effects on these types of achievement performance (e.g., learning tasks requiring flexible thinking such as convergent thinking) rather than subjects that require more analytic processing (e.g., mathematics). On the basis of our findings, future research should examine the boundary condition of when exercise enhances academic performance and when it does not. It is worth noting that we did not find a statistically significant effect of an exercise intervention on the subjective feeling of interest for the class itself. Previous studies indicated that a subjective feeling of interest is mainly driven by factors related to task content (e.g., task novelty; prior knowledge about the task)^54^. As exercising has nothing to do with the lesson content, our findings were consistent with these theoretical positions and may suggest the specificity of the beneficial effects of physical exercise in educational settings.

We did not find a significant effect of an exercise intervention on cognitive engagement. This was unexpected because cognitive engagement (i.e., sleep frequency and mind-wandering) can be considered as the downstream manifestation of emotional engagement (i.e., vigour and fatigue), for which we found the effects of exercise. Emotional engagement variables were positively correlated with cognitive engagement variables in our data (Table 2). One potential explanation was that the effects of exercise performed in this study on cognitive engagement may be weaker than those on emotional engagement, and the design may not have been sensitive enough to detect such small effects. From another perspective, exercise intensity might explain these effects. Our study focused on the potential beneficial effects of low-intensity exercise^30,47^. However, most previous studies that showed positive effects on cognitive engagement such as executive function were associated with moderate- to high-intensity exercise^20,55^. To date, there is limited evidence available concerning such effects of low-intensity exercise^30^. The results of this study may suggest that, when the intensity is low, exercise is more likely to have positive effects on emotions than on cognitive aspects. Another possibility is that mind-wandering and sleep frequency were subject to person-specific factors, while the resulting large individual differences may have masked the effects of exercise. It is also possible that retrospective measurement of mind-wandering and sleep frequency during each class suffered from subjective bias, which may have distorted the results. Future studies should examine whether exercise can facilitate cognitive engagement with a larger sample using more reliable measurements.

There were some limitations in the study. First, we did not include a pre-lesson motivation assessment. As a result, although our experimental design affords a relatively strong causal inference due to the (pseudo) random assignment of the conditions to the classes, it was not possible to examine whether there was an increase or decrease in self-reported motivation during class when students were engaged in physical exercise. The second limitation is the generalisability of the findings. We tested the hypothesis using a convenient sample of university students in one particular psychology class. Future studies should replicate these findings in various academic settings, establishing the robustness of the effects of exercise. Examining the generalisability of the findings is an important step in establishing such exercise activities as an effective intervention strategy in educational settings. Moreover, while we explained the effects of exercise might be mediated by physiological changes, we did not assess arousal or any physiological measures. Future research should incorporate measures of physiological changes, such as plasma measures, pupil diameters, and saliva alpha-amylase, which are non-invasive/peripheral measures of the locus coeruleus activity^56,57^.

Overall, our study is one of only a few studies that have examined the effects of exercise on certain aspects of motivation in educational settings despite some limitations. The previous studies have examined the beneficial effects of exercise on academic performance, but the underlying mechanisms behind the effects of exercise have been less clearly understood. In the current study, we observed that low-intensity exercise during class had a beneficial effect on emotional engagement as it resulted in high vigour and low fatigue. Given the importance of emotional engagement for achievement, our results may provide an insight into the mechanisms underlying the relationship between exercise and academic performance. Our adopted intervention can be incorporated into various educational settings in a straightforward manner (i.e., brief and low-intensity exercise). Thus, we hope that our findings will provide educational practitioners with a simple and useful strategy to motivate students.

## Methods

### Participants

This study was conducted as part of a weekly psychology class in a private university in Japan in 2018. They received an explanation of the study and provided written informed consent in the first week. Ultimately, 149 students participated. The study was approved by the institutional review board of Doshisha University Faculty of Psychology Research Ethics Committee.

The class was conducted in a standard lecture format, and its content was related to human motivation. We explained to students that the purpose of the study was to examine the effect of energising activities on their motivation towards class, and that participants would engage in two types of activities each time. We informed them that both types of activities would result in refreshment. In total, the intervention was conducted over nine weeks (one lesson per week) of the 15-week class, with four lessons assigned to the exercise condition and five to the control condition (i.e., a within-subjects design with repeated assessments). The order of the conditions was pseudo-randomly determined (i.e., alternated) but remained the same across participants as they all attended the same class. Specifically, all participants received the exercise and control class in the following order: control class, exercise class, control class, exercise class, control class, exercise class, control class, exercise class, and control class. Participants who provided inconsistent IDs (and thus did not provide longitudinal information) were excluded from analysis. For each time point, participants who missed the intervention either because they were absent or late to class were coded as missing. Furthermore, participants who attended the lessons only once for the 9-week intervention period were excluded. In total, we obtained 445 observations for the exercise condition and 565 for the control condition from 114 undergraduate students (men = 31, women = 80, unknown = 3, mean age = 20.46 years, age SD = 1.02).

### Procedure

The lecture lasted 90 min; in it, the instructor reviewed the contents of a lesson from the previous week for the first 20 min, followed by either the exercise or the control activity for three minutes. The intervention was followed by a lecture ~1-h long. During the activities, the instructor handed out the attendance record to everyone, so the activities were incorporated as a short break between the review section and the main part of the class. This means that the exercise was not an abrupt interruption of the class, but rather a natural transition to the main lesson content that day. This procedure was the same for all nine lessons. In the exercise condition, participants engaged in a three-minute slow aerobics exercise, following the expert-created DVD^44^. The slow aerobics activity consisted of a combination of three movements. The first movement consisted of students extending their arms backwards with their toes facing inward, then putting their hands together in front of their chest. In the second movement, participants bent their arms in front of their chest with their legs open to waist width; then, they shook their hips and hands while extending their hands overhead. In the third movement, students’ open arms were wrapped around their bodies, while their upper body was twisted. At this time, participants put their weight on one leg and raised the heel on the side they were twisting towards. Then, they put their weight on the opposite leg and wrapped their arms around the other side of their body. Participants performed these movements in time with the music while following the DVD. Participants practised these body movements in the first two lessons of the exercise condition and then combined all the movements in the last two lessons. In the control condition, we asked participants to watch a video for three minutes. The video displayed an underwater scene with sea creatures such as dolphins or whales, which was slightly altered every time for the five lessons in the control condition. We used the same background music for both conditions. At the end of the lesson, participants also completed a self-reported questionnaire assessing emotional and cognitive engagements. Supplementary Table 2 provides a report on the exercise programmes that we adopted in line with the Consensus on Exercise Reporting Template guidelines^58^, which is an internationally endorsed guideline for the reporting of exercise programmes across all evaluative study designs^58^.

### Materials

Below we list all the dependent variables that we assessed in the current examination. Each variable was assessed by a maximum of three items to reduce the load on participants. Items with the original version were slightly modified as was appropriate to the context of the class.

- Vigour, fatigue, and depression/dejection mood states: We used subscales from the Japanese version of the Profile of Mood States^59^ to measure the moods experienced at the time of the assessment (e.g., ‘To what degree do you feel this mood?’). Each subscale was assessed with two items, each rated on a scale of 1 (‘*not at all*’) to 5 (‘*extremely*’).
- Interest: We assessed interest in lesson content with three items taken from those used in Tanaka and Murayama (2014)^60^ (e.g., ‘Today’s class was interesting’). The items were rated on a scale of 1 (‘*not at all true*’) to 7 (‘*extremely true*’).
- Sleep frequency: We divided the 90-min class into 15-min blocks, asking participants to retrospectively report whether they fell asleep within each time frame (i.e., ‘Check the number corresponding to each of the six time periods to indicate if you were asleep during that period’). We summed the number of time bins checked and used this as an index of sleep frequency. The score ranged from 0 to 6.
- Mind-wandering: We assessed the degree of mind-wandering during class following items used in Killingsworth and Gilbert^61^ (i.e., ‘During today’s class, how much have you been thinking about something unrelated to course content?’). This item was rated on a scale of 1 (‘*not at all*’) to 10 (‘*most of the time*’).

We also assessed other variables that asked about participants’ state and activities before the intervention: i.e., amount of sleep, physical activities, and caffeine intake up to two hours before class. However, we did not analyse these variables. All analyses were performed using R software (version 3.6.1).

### Reporting summary

Further information on research design is available in the Nature Research Reporting Summary linked to this article.

## Supplementary information

Supplemental material

Reporting Summary

## Data Availability

The datasets generated during and analysed during this study are not publicly available because not all research on our project have been completed. They are available from the corresponding author on reasonable request.

## References

[1] Haskell WL (2007). Physical activity and public health: updated recommendation for adults from the American College of Sports Medicine and the American Heart Association. Circulation.

[2] Janssen I, LeBlanc AG (2010). Systematic review of the health benefits of physical activity and fitness in school-aged children and youth. Int. J. Behav. Nutr. Phys. Act..

[3] Childs E, de Wit H (2014). Regular exercise is associated with emotional resilience to acute stress in healthy adults. Front. Physiol..

[4] De Moor MHM, Beem AL, Stubbe JH, Boomsma DI, De Geus EJC (2006). Regular exercise, anxiety, depression and personality: a population-based study. Prev. Med..

[5] Mikkelsen K, Stojanovska L, Polenakovic M, Bosevski M, Apostolopoulos V (2017). Exercise and mental health. Maturitas.

[6] Barbosa A (2020). Physical activity and academic achievement: an umbrella review. Inter. J. Environ. Res. Public Health.

[7] Donnelly JE (2016). Physical activity, fitness, cognitive function, and academic achievement in children: a systematic review. Med. Sci. Sports Exerc..

[8] Rasberry CN (2011). The association between school-based physical activity, including physical education, and academic performance: a systematic review of the literature. Prev. Med..

[9] Bedard C, St John L, Bremer E, Graham JD, Cairney J (2019). A systematic review and meta-analysis on the effects of physically active classrooms on educational and enjoyment outcomes in school age children. PLOS ONE.

[10] Erwin H, Fedewa A, Beighle A, Ahn S (2012). A quantitative review of physical activity, health, and learning outcomes associated with classroom-based physical activity interventions. J. Appl. Sch. Psychol..

[11] Sember V, Jurak G, Kovač M, Morrison SA, Starc G (2020). Children’s physical activity, academic performance, and cognitive functioning: a systematic review and meta-analysis. Front. Public Health.

[12] Martin, A., Saunders, D. H., Shenkin, S. D., & Sproule, J. Lifestyle intervention for improving school achievement in overweight or obese children and adolescents. *Cochrane Database Syst. Rev*. **3** (2014). 10.1002/14651858.CD009728.pub2 24627300

[13] Masini A (2020). Evaluation of school-based interventions of active breaks in primary schools: a systematic review and meta-analysis. J. Sci. Med. Sport..

[14] Watson A, Timperio A, Brown H, Best K, Hesketh KD (2017). Effect of classroom-based physical activity interventions on academic and physical activity outcomes: a systematic review and meta-analysis. Int. J. Behav. Nutr. Phys. Act..

[15] Vazou S, Gavrilou P, Mamalaki E, Papanastasiou A, Sioumala N (2012). Does integrating physical activity in the elementary school classroom influence academic motivation. Int. J. Sport Exerc. Psychol..

[16] Murayama K, Pekrun R, Lichtenfeld S, Vom Hofe R (2013). Predicting long‐term growth in students’ mathematics achievement: the unique contributions of motivation and cognitive strategies. Child Dev..

[17] Reeve, J. M. *Understanding Motivation and Emotion,* 7th edn. (Wiley, 2018).

[18] Garcia D, Archer T, Moradi S, Andersson-Arntén AC (2012). Exercise frequency, high activation positive affect, and psychological well-being: beyond age, gender, and occupation. Psychology.

[19] Kennedy MM, Newton M (1997). Effect of exercise intensity on mood in step aerobics. J. Sports Med. Phys. Fit..

[20] Basso JC, Suzuki WA (2017). The effects of acute exercise on mood, cognition, neurophysiology, and neurochemical pathways: a review. Brain Plast..

[21] Greenwood BN (2019). The role of dopamine in overcoming aversion with exercise. Brain Res..

[22] Dishman RK, Renner KJ, White-Welkley JE, Burke KA, Bunnell BN (2000). Treadmill exercise training augments brain norepinephrine response to familiar and novel stress. Brain Res. Bull..

[23] Pagliari R, Peyrin L (1995). Norepinephrine release in the rat frontal cortex under treadmill exercise: a study with microdialysis. J. Appl. Physiol..

[24] Dremencov E (2017). Effect of physical exercise and acute escitalopram on the excitability of brain monoamine neurons: in vivo electrophysiological study in rats. Int. J. Neuropsychopharmacol..

[25] Greiwe JS, Hickner RC, Shah SD, Cryer PE, Holloszy JO (1999). Norepinephrine response to exercise at the same relative intensity before and after endurance exercise training. J. Appl. Physiol..

[26] Schmid A (1998). Free plasma catecholamines in spinal cord injured persons with different injury levels at rest and during exercise. J. Auton. Nerv. Syst..

[27] Wipfli B, Landers D, Nagoshi C, Ringenbach S (2011). An examination of serotonin and psychological variables in the relationship between exercise and mental health. Scand. J. Med. Sci. Sports.

[28] Ainley, M. Being and feeling interested: transient state, mood, and disposition. in *Mood in education* (ed. Schutz, P. A. &. Pekrun, R.) 57–73 (Academic Press, Cambridge, MA, 2007).

[29] Hidi S (2006). Interest: a unique motivational variable. Educ. Res. Rev..

[30] Byun K (2014). Positive effect of acute mild exercise on executive function via arousal-related prefrontal activations: an fNIRS study. Neuroimage.

[31] Aston-Jones G, Bloom FE (1981). Activity of norepinephrine-containing locus coeruleus neurons in behaving rats anticipates fluctuations in the sleep-waking cycle. J. Neurosci..

[32] Aston-Jones G, Cohen JD (2005). An integrative theory of locus coeruleus-norepinephrine function: adaptive gain and optimal performance. Annu. Rev. Neurosci..

[33] Mather M, Clewett D, Sakaki M, Harley CW (2016). Norepinephrine ignites local hotspots of neuronal excitation: how arousal amplifies selectivity in perception and memory. Behav. Brain Sci..

[34] Luque-Casado A (2016). Differences in sustained attention capacity as a function of aerobic fitness. Med. Sci. Sports Exerc..

[35] Fredricks JA, Blumenfeld PC, Paris AH (2004). School engagement: potential of the concept, state of the evidence. Rev. Educ. Res..

[36] Smallwood J, Fishman DJ, Schooler JW (2007). Counting the cost of an absent mind: mind wandering as an underrecognized influence on educational performance. Psychon. Bull. Rev..

[37] Smallwood J, Schooler JW (2006). The restless mind. Psychol. Bull..

[38] Risko EF, Anderson N, Sarwal A, Engelhardt M, Kingstone A (2012). Everyday attention: variation in mind wandering and memory in a lecture. Appl. Cogn. Psychol..

[39] Rodrigues RND, Viegas CA, Abreu e Silva AA, Tavares P (2002). Daytime sleepiness and academic performance in medical students. Arq. Neuropsiquiatr..

[40] Edens KM (2006). The relationship of university students’ sleep habits and academic motivation. NASPA J..

[41] Chen A, Yan J, Yin H, Pan C, Chang Y (2014). Effects of acute aerobic exercise on multiple aspects of executive function in preadolescent children. Psychol. Sport Exerc..

[42] Ferris LT, Williams JS, Shen CL (2007). The effect of acute exercise on serum brain-derived neurotrophic factor levels and cognitive function. Med. Sci. Sports Exerc..

[43] Smith PJ (2010). Aerobic exercise and neurocognitive performance: a meta-analytic review of randomized controlled trials. Psychosom. Med..

[44] Soya, M. In *Enhance Brain Fitness: Slow Aerobic* (ed. Japan Aerobic Federation) 1–77 (NHK Publishing, Inc., 2018).

[45] Burke SM, Carron AV, Eys MA, Ntoumanis N, Estabrooks PA (2006). Group versus individual approach? A meta-analysis of the effectiveness of interventions to promote physical activity. Sport Exerc. Psychol. Rev..

[46] Jones F, Harris P, Waller H, Coggins A (2005). Adherence to an exercise prescription scheme: The role of expectations, self-efficacy, stage of change and psychological well-being. Br. J. Health Psychol.

[47] Otsuka T (2016). Effects of acute treadmill running at different intensities on activities of serotonin and corticotropin-releasing factor neurons, and anxiety-and depressive-like behaviors in rats. Behavioural Brain Res..

[48] Kenny DA, La Voie L (1985). Separating individual and group effects. J. Personal. Soc. Psychol..

[49] Schielzeth H (2020). Robustness of linear mixed‐effects models to violations of distributional assumptions. Methods Ecol. Evol..

[50] Usami S, Murayama K (2018). Time-specific errors in growth curve modeling: type-1 error inflation and a possible solution with mixed-effects models. Multivar. Behav. Res..

[51] Brauer M, Curtin JJ (2018). Linear mixed-effects models and the analysis of nonindependent data: a unified framework to analyze categorical and continuous independent variables that vary within-subjects and/or within-items. Psychol. Methods.

[52] Maeda JK, Randall LM (2003). Can academic success come from five minutes of physical activity?. Brock Educ. J..

[53] Nadler RT, Rabi R, Minda JP (2010). Better mood and better performance: learning rule-described categories is enhanced by positive mood. Psychol. Sci..

[54] Renninger, K. A., & Hidi, S. E. *The Power of Interest for Motivation and Engagement* (Routledge, 2016).

[55] Aguirre-Loaiza H (2019). Effect of acute physical exercise on executive functions and emotional recognition: analysis of moderate to high intensity in young adults. Front. Psychol..

[56] Joshi S, Li Y, Kalwani RM, Gold JI (2016). Relationships between pupil diameter and neuronal activity in the locus coeruleus, colliculi, and cingulate cortex. Neuron.

[57] Nater UM, Rohleder N (2009). Salivary alpha-amylase as a non-invasive biomarker for the sympathetic nervous system: current state of research. Psychoneuroendocrinology.

[58] Slade SC (2016). Consensus on exercise reporting template (CERT): modified Delphi study. Phys. Ther..

[59] McNair, D. M., Lorr, M., & Droppleman, L. F. *Manual for the Profile of Mood States (POMS)* (Educational and Industrial Testing Service, 1971).

[60] Tanaka A, Murayama K (2014). Within-person analyses of situational interest and boredom: interactions between task-specific perceptions and achievement goals. J. Educ. Psychol..

[61] Killingsworth MA, Gilbert DT (2010). A wandering mind is an unhappy mind. Science.

