# Supplementary material for: The effect of low-intensity exercise on emotional and cognitive engagement in the classroom
Source: NPJ Sci Learn. 2022 May 26;7:9. doi: 10.1038/s41539-022-00125-y (PMC9135685; doi:10.1038/s41539-022-00125-y)
Supplement: Supplementary file 1 — Supplemental material [file 41539_2022_125_MOESM1_ESM.pdf]

Supplementary Table 1 Results of linear mixed-effects models with the original model prior to addressing the convergence and singularity problems

	Vigour			Fatigue			Depression			Interest			Sleep Frequency			Mind-wandering		
	B	*t*	*p* val.	*B*	*t*	*p* val.	*B*	*t*	*p* val.	*B*	*t*	*p* val.	*B*	*t*	*p* val.	*B*	*t*	*p* val.
Intercept	2.85	43.32	.00	2.72	37.74	.00	1.54	29.34	.00	5.15	69.45	.00	0.30	5.57	.00	3.81	24.88	.00
Condition	0.10	3.84	.00	-0.09	-2.70	.01	-0.04	-1.68	.10	0.04	1.59	.12	-0.06	-1.37	.21	-0.04	-0.74	.45
Time	-0.02	-1.69	.09	0.00	0.07	.94	-0.01	-1.34	.18	-0.06	-4.83	.00	0.02	1.04	.33	-0.01	-0.42	.68

Note. The code of the condition was exercise condition = 1 and control condition = -1. *p* val. = two-tailed *P* value.

Supplementary Table 2 Consensus on Exercise Report Template guidelines of the current study

Item Category	Item No.	Abbreviated Item Description
**WHAT**: materials	1	**Type of exercise equipment**<br><br>We screened a DVD[1] created by an expert on a projector.
**WHO**: provider	2	**Qualifications, teaching/supervising expertise, and/or training of the exercise instructor**<br><br>The lecturer in charge of the class received expert guidance, but neither expert nor lecturer supervised students directly. Participants performed exercise according to the DVD instruction[1]. In the video, professionals provided exercise guidance step by step.
**HOW**: delivery	3	**Whether exercises are performed individually or in a group**<br><br>Exercises were performed in a group in a classroom. The program was conducted in a psychology class with 149 students participating.
	4	**Whether exercises are supervised or unsupervised**<br><br>As mentioned above (item 2), neither expert nor lecturer supervised participants directly. Participants followed the

		leaders on the video to exercise but were not directly supervised regarding their proficiency.
	5	**Measurement and reporting of adherence to exercise**<br><br>Participants indicated whether they had participated in the exercise (or watched the video in the control condition) by answering a series of items on the questionnaire administered at the end of the lesson.
	6	**Details of motivation strategies**<br><br>No special motivation strategies for exercise were employed. However, we explained to the students that the purpose of the study was to examine the effect of energizing activities on motivation towards class. In addition, the exercise was as brief as three minutes and low intensity in some instances, so it was relatively easy to perform.
	7	**Decision rules for progressing the exercise program**<br><br>The intervention was conducted over nine weeks (one lesson per week) of the 15-week class, with four lessons assigned to the exercise condition and five to the control condition. (We did not run the experiments for the other weeks.) Exercise consisted of a combination of three movements. Participants practiced three movements during the first two lessons of the exercise condition. Subsequently, they combined all the

		movements in the last two lessons.
	8	**Each exercise is described so that it can be replicated (e.g., illustrations, photographs)**<br><br>We adopted slow aerobic exercise which comprised three dynamic upper-body stretches: (a) flare-twist, (b) furifuri-guppa, and (c) nobi-yura.<br>(a) Flare-twist consists of twisting the upper body.<br>(b) The movement furifuri-guppa involves pulling the elbows back and then clapping the hands while shaking the waist from side to side.<br>(c) Nobi-yura is waving the arms as if wiping windows while shaking the waist from side to side.<br>Participants performed these movements in time with the music while following the DVD[1]. Photographs were provided from previous studies that adopted an exercise programme that incorporated the same movements[2,3].
	9	**Content of any home program component**<br><br>There were no home program components.
	10	**Non-exercise components**<br><br>In the control condition, we asked participants to watch a video for three minutes. The video displayed an underwater scene with sea creatures such as dolphins or whales, which

		was slightly altered every time for the five lessons in the control condition. We used the same background music for both conditions.
	11	**How adverse events that occur during exercise are documented and managed**<br><br>Since we adopted low-intensity exercise, it was presumed that adverse events were unlikely to occur. However, if the participant felt discomfort, they could stop participating in the experiment at any time. No participants reported adverse events or discomfort.
**WHERE**: location	12	**Setting in which exercises are performed**<br><br>This study was conducted as part of a weekly psychology class offered at a private university.
**WHEN, HOW MUCH**: dosage	13	**Detailed description of the exercises (e.g., sets, repetitions, duration, intensity)**<br><br>We adopted low intensity (corresponding to ‘very light’ in American College of Sports Medicine guidelines[4]) and three-minute versions of a slow aerobic exercise program[1]. The intervention was carried out during class. The lecture lasted 90 minutes. During lecture, the instructor reviewed the contents of a lesson from the previous week for the first 20 minutes, followed by either exercise or the control activity

		for three minutes. The intervention was followed by a lecture approximately one-hour long. The same procedure was performed for all nine lessons. As mentioned above (item 7), participants practiced three movements in the first two lessons of the exercise condition. They combined all the movements in the last two lessons.
	14	**Whether exercises are generic ('one size fits all') or tailored to the individual**<br><br>The exercise program we adopted is generic, but can be flexibly adjusted to different situations and individuals[1].
**HOW WELL**: planned, actual	15	**Decision rule that determines the starting level for exercise**<br><br>We did not set an individual starting level for exercise. All participants started with the same versions of the three movements.
	16	**Whether the exercise intervention is delivered and performed as planned**<br><br>The class lecturer checked that the students were performing the exercise (or watching the video) to determine whether the intervention was being delivered and performed in the intended manner.

Note. Detailed description of the exercise intervention applied in the study 'The effect of low-

intensity exercise on emotional and cognitive engagement in the classroom', reporting follows the Consensus on Exercise Report Template guidelines[5].

Supplementary References

1. Soya, M. Enhance brain fitness: Slow aerobic (ed. Japan Aerobic Federation). (NHK Publishing, Inc., Tokyo, 2018).
2. Hyodo, K., Jindo, T., Suwabe, K., Soya, H., & Nagamatsu, T. (2019). Acute effects of light-intensity, slow-tempo aerobic dance exercise on mood and executive function in older adults. *Bull. Phys. Fit. Res. Inst.* **117**, 8–16.
3. Hyodo, K., Suwabe, K., Yamaguchi, D., Soya, H., & Arao, T. (2021). Comparison between the effects of continuous and intermittent light-intensity aerobic dance exercise on mood and executive functions in older adults. *Front. Aging Neurosci.* **13**.
4. Garber, C. E., Blissmer, B., Deschenes, M. R., Franklin, B. A., Lamonte, M. J., Lee, I. M., ... & Swain, D. P. (2011). Quantity and quality of exercise for developing and maintaining cardiorespiratory, musculoskeletal, and neuromotor fitness in apparently healthy adults: Guidance for prescribing exercise. *Med. Sci. Sports Exerc.* **43**(7), 1334–59.
5. Slade, S. C., Dionne, C. E., Underwood, M., Buchbinder, R., Beck, B., Bennell, K., ... & White, C. (2016). Consensus on exercise reporting template (CERT): Modified Delphi study. *Phys. Ther.* **96**(10), 1514–1524.
