# Supplementary material for: The effect of low-intensity exercise on emotional and cognitive engagement in the classroom
Source: NPJ Sci Learn. 2022 May 26;7:9. doi: 10.1038/s41539-022-00125-y (PMC9135685; doi:10.1038/s41539-022-00125-y)
Supplement: Supplementary file 2 — Reporting Summary [file 41539_2022_125_MOESM2_ESM.pdf]

nature portfolio

Corresponding author(s): Ayame Tamura

Last updated by author(s): Jun 28, 2021

Reporting Summary

Nature Portfolio wishes to improve the reproducibility of the work that we publish. This form provides structure for consistency and transparency in reporting. For further information on Nature Portfolio policies, see our Editorial Policies and the Editorial Policy Checklist.

Statistics

For all statistical analyses, confirm that the following items are present in the figure legend, table legend, main text, or Methods section.

n/a	Confirmed	
☐	☒	The exact sample size (n) for each experimental group/condition, given as a discrete number and unit of measurement
☐	☒	A statement on whether measurements were taken from distinct samples or whether the same sample was measured repeatedly
☐	☒	The statistical test(s) used AND whether they are one- or two-sided<br>*Only common tests should be described solely by name; describe more complex techniques in the Methods section.*
☒	☐	A description of all covariates tested
☐	☒	A description of any assumptions or corrections, such as tests of normality and adjustment for multiple comparisons
☐	☒	A full description of the statistical parameters including central tendency (e.g. means) or other basic estimates (e.g. regression coefficient) AND variation (e.g. standard deviation) or associated estimates of uncertainty (e.g. confidence intervals)
☐	☒	For null hypothesis testing, the test statistic (e.g. F, t, r) with confidence intervals, effect sizes, degrees of freedom and P value noted<br>*Give P values as exact values whenever suitable.*
☒	☐	For Bayesian analysis, information on the choice of priors and Markov chain Monte Carlo settings
☐	☒	For hierarchical and complex designs, identification of the appropriate level for tests and full reporting of outcomes
☐	☒	Estimates of effect sizes (e.g. Cohen's d, Pearson's r), indicating how they were calculated

Our web collection on statistics for biologists contains articles on many of the points above.

Software and code

Policy information about availability of computer code

Data collection	A series of questionnaires were collected online using Qualtrics software.
Data analysis	All analyses were performed by R software (version 3.6.1).

For manuscripts utilizing custom algorithms or software that are central to the research but not yet described in published literature, software must be made available to editors and reviewers. We strongly encourage code deposition in a community repository (e.g. GitHub). See the Nature Portfolio guidelines for submitting code & software for further information.

Data

Policy information about availability of data

All manuscripts must include a data availability statement. This statement should provide the following information, where applicable:

- Accession codes, unique identifiers, or web links for publicly available datasets
- A description of any restrictions on data availability
- For clinical datasets or third party data, please ensure that the statement adheres to our policy

The datasets generated during and analysed during the current study are not publicly available because not all research on our project have been completed. They are available from the corresponding author on reasonable request.

Field-specific reporting

Please select the one below that is the best fit for your research. If you are not sure, read the appropriate sections before making your selection.

☐ Life sciences ☒ Behavioural & social sciences ☐ Ecological, evolutionary & environmental sciences

For a reference copy of the document with all sections, see nature.com/documents/nr-reporting-summary-flat.pdf

Behavioural & social sciences study design

All studies must disclose on these points even when the disclosure is negative.

Study description	The data in this study are qualitative.
Research sample	Research sample is 114 undergraduates of Doshisha university (men = 31, women = 80, unknown = 3, mean age = 20.46 years, age SD = 1.02). We aimed to examine the effect of exercise on motivation in an educational setting, but it has limitations in terms of generalizability (discussed in the manuscript).
Sampling strategy	We used convenience sampling procedure. We first decided a university class to be used in the intervention, and then recruited all the students who got registered to the class. There was no optimal stopping in the sampling procedure. Using the method proposed by Murayama, Usami, and Sakaki (2020), sensitivity analysis shows that the sample size is sufficient to detect the effect size of Cohen's d = 0.34 at 95% power (alpha = 0.05, two-tailed, no random lesson effect was supposed). Note that this effect size represents the intervention effects scaled by the individual differences of the intervention effects (Murayama et al., 2019, https://osf.io/6cer3/).
Data collection	The experiment was conducted during class and there were only participants and instructors in the classroom. Participants completed questionnaire on the website (Qualtrics) using their smartphones. The study was single-blind because the experimenter understood the study outline.
Timing	The data collection started on October 15, 2018 and ended on January 14, 2019.
Data exclusions	The data which could not been tracked their ID were excluded from the analysis. Participants who missed the intervention either because they were absent or late to class were coded as missing. Furthermore, participants who attended the lessons only once for the 9-week intervention period were excluded. We excluded 459 observations for the exercise condition and 565 for the control condition.
Non-participation	6 students declined to participate in the informed consent conducted during the first experiment. Participation in the experiment was free in the subsequent classes, but the reasons for non-participation in the experiment could not be determined as absent, late, or willingness to decline.
Randomization	Participants experienced both experimental and control conditions because we used in-participant design. The order of the conditions was pseudo-randomly determined.

Reporting for specific materials, systems and methods

We require information from authors about some types of materials, experimental systems and methods used in many studies. Here, indicate whether each material, system or method listed is relevant to your study. If you are not sure if a list item applies to your research, read the appropriate section before selecting a response.

Materials & experimental systems

n/a	Involved in the study	
☒	☐	Antibodies
☒	☐	Eukaryotic cell lines
☒	☐	Palaeontology and archaeology
☒	☐	Animals and other organisms
☐	☒	Human research participants
☒	☐	Clinical data
☒	☐	Dual use research of concern

Methods

n/a	Involved in the study	
☒	☐	ChIP-seq
☒	☐	Flow cytometry
☒	☐	MRI-based neuroimaging

Human research participants

Policy information about studies involving human research participants

Population characteristics	See above.

Recruitment	Participants were recruited from the lesson described in the manuscript. We first decided a university class to be used in the intervention, and then recruited all the students who got registered to the class.
Ethics oversight	The study was approved by the institutional review board of Doshisha University Faculty of Psychology Research Ethics Committee (Title: Research on the effect of refreshing activities in the classroom, Number: KH57).

Note that full information on the approval of the study protocol must also be provided in the manuscript.
